# Editorial: The mechanism of metabolic immune microenvironment, inflammation and blood milk barrier in mastitis

**DOI:** 10.3389/fimmu.2023.1213826

**Published:** 2023-06-01

**Authors:** Wenjin Guo, Shoupeng Fu, Juxiong Liu, Yi Zhu

**Affiliations:** ^1^ State Key Laboratory for Diagnosis and Treatment of Severe Zoonotic Infectious Diseases, Key Laboratory for Zoonosis Research of the Ministry of Education, Institute of Zoonosis, and College of Veterinary Medicine, Jilin University, Changchun, China; ^2^ Children Nutrition Research Center, Baylor College of Medicine, Houston, TX, United States

**Keywords:** mastitis, metabolic immune microenvironment, blood milk barrier, inflammation, signaling pathway

Dairy cow mastitis is a bottleneck problem that troubles the development of the world milk industry, with economic losses caused by dairy cow mastitis reaching billions of dollars annually. At present, antibiotics are mainly used in the clinical treatment of dairy cow mastitis. Although antibiotics can kill bacteria in the mammary gland, it is difficult to control the inflammatory response in the mammary gland. Inflammatory reactions in the mammary gland can lead to various adverse outcomes, such as mammary gland aging, fibrosis, or disruption of the blood milk barrier. Therefore, the treatment of dairy cow mastitis should be multi angle and multi method, not only to control bacterial reproduction, but also to intervene the immune microenvironment, blood milk barrier and inflammatory reaction in the mammary gland.

Numerous studies have shown that mammary epithelial cells are the most important lactating cells, and the function and health of mammary epithelial cells are one of the important factors that restrict the lactation ability of dairy cows (1). From the perspective of maintaining the health of dairy cows’ mammary glands, reducing damage to lactating cells, ensuring the health of dairy cow mammary glands, increasing lactation lifespan and milk production are important research directions in this field. Current research also indicates that dairy cow mastitis is one of the key factors leading to mammary gland damage and reduced lactation cells in dairy cows, and the occurrence and development of dairy cow mastitis are mainly caused by the combined action of exogenous and endogenous factors (2). In recent years, studies have found that the accumulation of damage related molecules in the mammary gland caused by mastitis can cause long-term and long-lasting damage to the mammary gland. Although antibiotic therapy developed for bacterial infections can inhibit pathogens in the mammary gland, but ignoring damage related molecules in the mammary gland may lead to deeper mammary gland damage and even lead to the death of dairy cows.

The researchers in this topic conducted in-depth discussions on the inflammatory reaction, blood milk barrier and immune micro-environment in the mammary gland, and found that the formation of neutrophil extracellular trap net in the blood of dairy cows with high somatic cell number and low somatic cell number is different, which may be a potential indicator of the risk prognosis of mastitis (Jiang et al.). Some studies have also found that Houttuynia essential oil could significantly alleviate mastitis, which is achieved by inhibiting the MAPK signaling pathway (Liu et al.). Formononetin and Menthol could also significantly inhibit inflammatory reactions in the mammary gland and enhance the blood milk barrier, but their mechanisms are completely different. Formonetin primarily exerts its anti-inflammatory function by inhibiting the NF-κB signaling pathway (Xiang et al.), while Menthol primarily exerts anti-inflammatory functions by activating the AMPK signaling pathway and autophagy (Liu et al.). And researchers have found that Menthol can enhance the blood milk barrier. The above research indicates that the occurrence and development of mastitis are mainly related to inflammatory damage in mammary epithelial cells, and how to alleviate this inflammatory damage is a key means to improve mastitis.

This topic mainly studies the immune microenvironment, blood milk barrier and inflammatory reaction in the process of mastitis. Many researchers discussed in this Research Topic and found a variety of natural products with anti-mastitis activity and molecular mechanisms related to the prognosis of mastitis.

## Author contributions

All authors listed have made a substantial, direct, and intellectual contribution to the work and approved it for publication.
